# Supplementary material for: Disease dynamics and potential mitigation among restored and wild staghorn coral, Acropora cervicornis
Source: PeerJ. 2014 Aug 28;2:e541. doi: 10.7717/peerj.541 (PMC4157300; doi:10.7717/peerj.541)
Supplement: Table S1 — Characteristics and scoring of changes noted in cells and tissues using light microscopic examination of A. cervicornis subsamples collected during this study. [file peerj-02-541-s001.docx]

| **Parameter Viewed at 100x or 250+x, Description of “Normal”** | **Numerical Score**  **Intensity or Severity Score** | | | | |
| --- | --- | --- | --- | --- | --- |
| **0 (No Change)** | **1** | **2** | **3** | **4** | **5** |
| **Low Magnification** | **Very Good** | **Good** | **Fair** | **Poor** | **Very Poor** |
| General Condition  0 = Excellent, similar to 1970s samples, thick epithelia and mesoglea, mucocytes not hypertrophied, highly cellular | Similar to 1970s samples, but epithelia and mesoglea not as thick, epidermal mucocytes slightly hypertrophied | Hypertrophy of epidermal mucocytes, intact epithelia and mesoglea, mesentery and filament architecture still normal | Hypertrophy of epidermal mucocytes, minimal to mild attenuation (atrophy) of epithelia and mesoglea noted | Loss of mucocytes, moderate attenuation of epithelia and mesoglea, mesentery and filament architecture degenerating | Severe attenuation of epithelia and mesoglea, loss of epitheliomuscular cells with vacuolation of mesogleal pleats necrosis and dissociation of mesenterial filaments, necrosis and lysing of epithelial cells |
| Zooxanthellae  0 = Gastrodermal cells packed with well-stained algal symbionts in surface body wall, tentacles; scattered algal symbionts deeper in gastrovascular canals and absorptive cells next to mesenterial filaments | Similar to 1970s samples, thick layer of well-stained algal symbionts in gastrodermis of surface body wall, tentacles, and scattered cells in gastrovascular canals and absorptive cells next to mesenterial filaments | Thick layer of well-stained algal symbionts, but not quite as abundant as in 1970s samples | Algal symbionts fewer in gastrodermis which is mildly attenuated (atrophied), most still stain appropriately | Single row of algal symbionts in surface body wall gastrodermis and markedly fewer in tentacle gastrodermis, some have lost acidophilic staining as proteins no longer produced or nucleus/cytoplasm lysed, vacuole enlarged compared to algal cell | No zooxanthellae present in cuboidal gastrodermal cells of colony (bleached) |
| **High Magnification** | **Minimal** | **Mild** | **Moderate** | **Marked** | **Severe** |
| Epidermal Mucocytes  0 = In 1970s sample, thin columnar cells, uniform distribution and not taller than ciliated supporting cells, pale mucus | Slightly hypertrophied, numerous, pale-staining frothy mucus | Many cells hypertrophied, abundant release of pale-staining mucus | Uneven appearance of mucocytes, some hypertrophied but some reduced in size and secretion, darker staining mucus | Some epidermal foci lack mucocytes entirely, attenuation (atrophy) of epidermis evident, darker staining and stringy mucus | Loss of many mucocytes, epidermis is attenuated to at least half of normal thickness or more, if mucus present it stains dark, thick |
| Cnidoglandular Band Epithelium Mucocytes  0 = Oral portion lacks mucocytes, increasing in number aborally, may be abundant with pale mucus; difficult to assess significance of appearance | Less than half the area of cnidoglandular band is mucocytes, but could be more depending on location along the filament, size of mucocytes variable | About half the area is mucocytes, some hypertrophied | About half the area is mucocytes, all hypertrophied | About three quarters of the area is mucocytes, mucus production reduced, some vacuolation present | Loss of mucocytes, vacuolation and necrosis of cells present |
| Degeneration of Cnidoglandular Bands  0 = Ciliated columnar cells, nematocytes, acidophilic granular gland cells, and mucocytes abundant (but varying with location), tall, thin columnar, contiguous, terminal bar well formed | Mild reduction in cell height | Cell height more reduced, mild loss of mucocytes or secretions | Attenuation (atrophy), loss of cells | Moderate attenuation of epithelium, some granular gland cells stain dark pink and are rounded, not columnar, terminal bar not contiguous, some pycnotic nuclei present, loss of cells by detachment and sloughing | Severe atrophy of epithelium, detachment from mesoglea and loss of cells, necrosis or apoptosis of remaining cells, no terminal bar present, loss of cilia |
| Dissociation of Cells on Mesenterial Filaments  0 = All cells intact and within normal limits, contiguous, thin columnar morphology, terminal bar present, cilia visible along apical surface | Minimal loss of cilia, but will not be present where mucocytes are predominant | Minimal to mild loss of cells, terminal bar has minute gaps indicating loss of ciliated cells | Attenuation (atrophy) of cells, vacuolation, reduced cilia, but filament still intact | Rounding up and loss of granular gland cells, some pycnotic nuclei present, cell loss evident, terminal bar gaps, terminal web (junctions) between cells lost, starting to spread apart along cnidoglandular band | Marked to severe separation of cells, most necrotic with pycnotic nuclei, vacuolated, lysing and loss of mucocytes, nematocysts, granular gland cells and ciliate columnar cells |
| Costal Tissue Loss  0 = Tissue covering costae intact, epidermis similar in thickness to epidermis of surface body wall with gastrodermis as it covers the costae, although this may vary with location and be thinner; calicodermis thick, pale to clear cytoplasm, or thinner with cytoplasmic extensions apically | Attenuation (atrophy) of epidermis, mesoglea, and calicodermis, but still intact over costae | Up to one-quarter of costae on corallite surfaces exposed due to loss of epithelia and mesoglea | Up to one-half of costae exposed | About three quarters of costae exposed | Most costae exposed or gaps in surface body wall, tissues atrophied |
| Calicodermis Condition  0 = Calicoblasts numerous, squamous but thick cytoplasm | Calicoblasts slightly reduced in height focally (more likely interior of colony) | About half of calicoblasts attenuated (atrophied), loss of proteins in cytoplasm | Most calicoblasts attenuated, fewer in number, spread out thinly on mesoglea, still cuboidal to columnar and active under surface body wall and in apical polyps | Most calicoblasts markedly atrophied, fewer in number, some separating from mesoglea | Surface body wall calicoblasts severely atrophied or vacuolated, detaching and sloughing, missing from mesoglea |
| WBD Bacterial Aggregates  0 = Not present in tissue (between mesoglea and calicodermis) lining gastrovascular canals | One bacterial aggregate found in tissue on slide (rare) | Two to five bacterial aggregates found in tissue on slide (occasional) | Density increases, 6 to 10 bacterial aggregates in tissue on slide (common) | Density increases, bacterial aggregates seen throughout tissue on slide (frequent) | Numerous bacterial aggregates present in any area of tissue, multiple adjacent aggregates, size 50–100 µm (abundant) |
| Epidermal RLOs  0 = Not present | One infected cell on oral disks or tentacles of polyps (rare) | Several infected cells on oral disks or tentacles of polyps, numerous mucocytes present (occasional) | About half of mucocytes infected on oral disks or tentacles of polyps, loss of some mucocytes (common), rare infected cells in actinopharynx epidermis | More than half of mucocytes infected on oral disks or tentacles of polyps, loss of mucocytes (frequent), increase in infected cells on actinopharynx epidermis | Nearly all remaining mucocytes infected (may have lost many as infected cells die and lyse), many infected cells in actinopharynx epidermis (abundant) |
| Filament RLOs  0 = Not present | One infected cell on cnidoglandular bands (rare) | Several infected cells on cnidoglandular bands present in tissue section (occasional) | Infected cells present on about half of sections through cnidoglandular bands (common), slight loss of mucocytes, a few infected mucocytes in gastrodermis lining gastrovascular canals (rare) | A few infected cells present on almost all sections through cnidoglandular bands (frequent), loss of mucocytes, more infected cells in gastrodermis lining gastrovascular canals (common) | Nearly all remaining mucocytes infected but many lost as infected cells die and lyse, mucocytes of gastrodermis or mesenteries infected (abundant) |
| **Gonad Staging**  **0 = None present** | **1** | **2** | **3** | **4** | **5** |
| Oocytes | Single germ cell surrounded by mesoglea in mesentery | Early oocyte, nucleus with distinct nucleolus but little development of lipid and protein in cytoplasm | Mid-development, uniform distribution of lipid droplets and protein granules, nucleus and cytoplasm enlarge | Mature, development of cortical granules and vitelline membrane, beginning to separate from mesoglea | Spawned, hole present where ovum released to gastrovascular cavity |
| Spermaries | Germ cells aggregate in mesoglea, forming one or a few clusters | Early spermaries, multiplication of germ cells, mitotic figures present | More spermaries present, spermatocytes undergo meiosis, spermatids fill lumen | Mature spermatozoa fill lumen, may still have earlier stages surrounding these, but eventually all change to spermatozoa | Spawned, remnants of spermatozoa endocytosed by absorptive gastrodermal cells on mesentery |
