# Supplementary material for: Disease dynamics and potential mitigation among restored and wild staghorn coral, Acropora cervicornis
Source: PeerJ. 2014 Aug 28;2:e541. doi: 10.7717/peerj.541 (PMC4157300; doi:10.7717/peerj.541)
Supplement: Table S2 — Frequency distributions of condition or severity/intensity scores for tissue parameters of apparently healthy and diseased A. cervicornis samples. Shaded cells show numbers of samples in each category sharing scores. For example, condition scores for epidermal mucocytes were clearly separated between the apparently healthy (none to moderate changes) and diseased samples (only marked to severe microscopic changes), whereas for mesenterial filament mucocytes, distinctions between healthy or diseased samples (based on gross appearance) were less apparent microscopically, as evidenced by their overlapping scores (shaded cells). [file peerj-02-541-s002.docx]

| **Parameter** | **Healthy**  **(number affected)** | **Diseased**  **(number affected** | **Parameter** | **Healthy**  **(number affected)** | **Diseased**  **(number affected)** |
| --- | --- | --- | --- | --- | --- |
| General Condition 100x |  |  | Zooxanthellae Condition 100x |  |  |
| 0 | 0 | 0 | 0 | 1 | 0 |
| 1 | 11 | 0 | 1 | 15 | 0 |
| 2 | 6 | 0 | 2 | 4 | 0 |
| 3 | 4 | 1 | 3 | 1 | 4 |
| 4 | 0 | 3 | 4 | 0 | 17 |
| 5 | 0 | 18 | 5 | 0 | 1 |
| Epidermal Mucocytes Condition |  |  | Mesenterial Filament Mucocytes |  |  |
| 0 | 0 | 0 | 0 | 0 | 0 |
| 1 | 7 | 0 | 1 | 3 | 0 |
| 2 | 12 | 0 | 2 | 5 | 1 |
| 3 | 2 | 0 | 3 | 7 | 1 |
| 4 | 0 | 5 | 4 | 4 | 5 |
| 5 | 0 | 17 | 5 | 2 | 15 |
| Degeneration Cnidoglandular Bands |  |  | Dissociation Mesenterial Filaments |  |  |
| 0 | 5 | 0 | 0 | 13 | 1 |
| 1 | 5 | 0 | 1 | 5 | 4 |
| 2 | 6 | 3 | 2 | 1 | 4 |
| 3 | 3 | 2 | 3 | 2 | 6 |
| 4 | 1 | 1 | 4 | 0 | 5 |
| 5 | 1 | 16 | 5 | 0 | 2 |
| Costal Tissue Loss |  |  | Calicodermis Condition |  |  |
| 0 | 13 | 0 | 0 | 0 | 0 |
| 1 | 8 | 1 | 1 | 12 | 0 |
| 2 | 0 | 3 | 2 | 8 | 0 |
| 3 | 0 | 5 | 3 | 0 | 3 |
| 4 | 0 | 4 | 4 | 1 | 9 |
| 5 | 0 | 9 | 5 | 0 | 10 |
| Epidermal RLOs |  |  | Filament RLOs |  |  |
| 0 | 0 | 0 | 0 | 0 | 0 |
| 1 | 0 | 0 | 1 | 0 | 1 |
| 2 | 2 | 0 | 2 | 6 | 8 |
| 3 | 12 | 9 | 3 | 14 | 8 |
| 4 | 7 | 13 | 4 | 1 | 3 |
| 5 | 0 | 0 | 5 | 0 | 2 |
