# Supplementary material for: Disease dynamics and potential mitigation among restored and wild staghorn coral, Acropora cervicornis
Source: PeerJ. 2014 Aug 28;2:e541. doi: 10.7717/peerj.541 (PMC4157300; doi:10.7717/peerj.541)
Supplement: Table S3 — Results of tests of significance for the data comparisons for each tissue parameter based on condition or severity/intensity scores. WBD Bacterial Aggregates is not included since none were found in any of the samples. ∗ = significantly different at the P ≤ 0.05. [file peerj-02-541-s003.docx]

| **Parameter** | **F-test** | **T-test**  **Variance-adjusted** | **Mann-Whitney U-test** | | |
| --- | --- | --- | --- | --- | --- |
|  | **Var 1 > Var 2** | **P value** | **Adjusted H** | **d.f.** | **P value** |
| **Apparently Healthy (n = 21) vs Diseased (n = 22)** | | | | | |
| General Condition 100x | 0.3508 | 3.0018E-17* | 31.88 | 1 | 1.64E-8* |
| Zooxanthellae 100x | 0.1494 | 1.2152E-20* | 32.95 | 1 | 9.45E-9* |
| Epidermal Mucocytes Condition | 0.9466 | 1.3631E-19* | 32.188 | 1 | 1.40E-8* |
| Mesenterial Filament Mucocytes | 0.1379 | 4.6363E-06* | 17.029 | 1 | 0.000037* |
| Degeneration Cnidoglandular Bands | 0.6488 | 2.1242E-08* | 22.602 | 1 | 1.99E-6* |
| Dissociation of Mesenterial Filaments | 0.0848 | 5.5893E-06* | 18.852 | 1 | 0.000014* |
| Costal Tissue Loss | 1.7459E-05* | 1.2021E-10* | 30.823 | 1 | 2.83E-8* |
| Calicodermis Condition | 0.3171 | 1.2481E-14* | 31.445 | 1 | 2.05E-8* |
| Epidermal RLOs | 0.4250 | 0.1645 | 0.898 | 1 | 0.343 |
| Filament RLOs | 0.005204* | 0.7672 | 0.069 | 1 | 0.793 |
| **Mitigation Treatment Successful (n = 6) vs Unsuccessful (n = 5)** | | | | | |
| General Condition 100x | 0.2835 | 0.1365 | 2.75 | 1 | 0.097 |
| Zooxanthellae 100x | 0.01097* | 0.5413 | 0.853 | 1 | 0.356 |
| Epidermal Mucocytes Condition | 0.7872 | 0.2768 | 0.833 | 1 | 0.361 |
| Mesenterial Filament Mucocytes | 0.03643* | 0.03124* | 6.685 | 1 | 0.009721* |
| Degeneration Cnidoglandular Bands | 0.004764* | 0.01880* | 5.685 | 1 | 0.017* |
| Dissociation of Mesenterial Filaments | 0.5784 | 0.8976 | 0.008371 | 1 | 0.927 |
| Costal Tissue Loss | 0.3994 | 0.3132 | 0.833 | 1 | 0.361 |
| Calicodermis Condition | 0.7088 | 0.8084 | 0.033 | 1 | 0.855 |
| Epidermal RLOs | 0.5801 | 0.21106 | 1.695 | 1 | 0.193 |
| Filament RLOs | 0.3532 | 0.7665 | 0.917 | 1 | 0.338 |
| **Characteristics of WBD (n = 9) vs RTL (n = 12)** | | | | | |
| General Condition 100x | 0.4899 | 0.8863 | 0.001293 | 1 | 0.971 |
| Zooxanthellae 100x | 0.3206 | 0.9705 | 0.00E0 | 1 | 1.0 |
| Epidermal Mucocytes Condition | 0.8670 | 0.2391 | 0.046 | 1 | 0.831 |
| Mesenterial Filament Mucocytes | 0.1475 | 0.5939 | 2.176 | 1 | 0.14 |
| Degeneration Cnidoglandular Bands | 0.7407 | 0.5078 | 1.937 | 1 | 0.164 |
| Dissociation of Mesenterial Filaments | 0.9369 | 0.7557 | 0.032 | 1 | 0.859 |
| Costal Tissue Loss | 0.9678 | 0.6419 | 0.153 | 1 | 0.696 |
| Calicodermis Condition | 0.9116 | 0.3742 | 0.00507 | 1 | 0.943 |
| Epidermal RLOs | 0.2791 | 0.02541* | 4.672 | 1 | 0.031* |
| Filament RLOs | 0.7776 | 0.6275 | 1.054 | 1 | 0.305 |
