# Supplementary figures and images for: Disease dynamics and potential mitigation among restored and wild staghorn coral, Acropora cervicornis
Source: PeerJ. 2014 Aug 28;2:e541. doi: 10.7717/peerj.541 (PMC4157300; doi:10.7717/peerj.541)

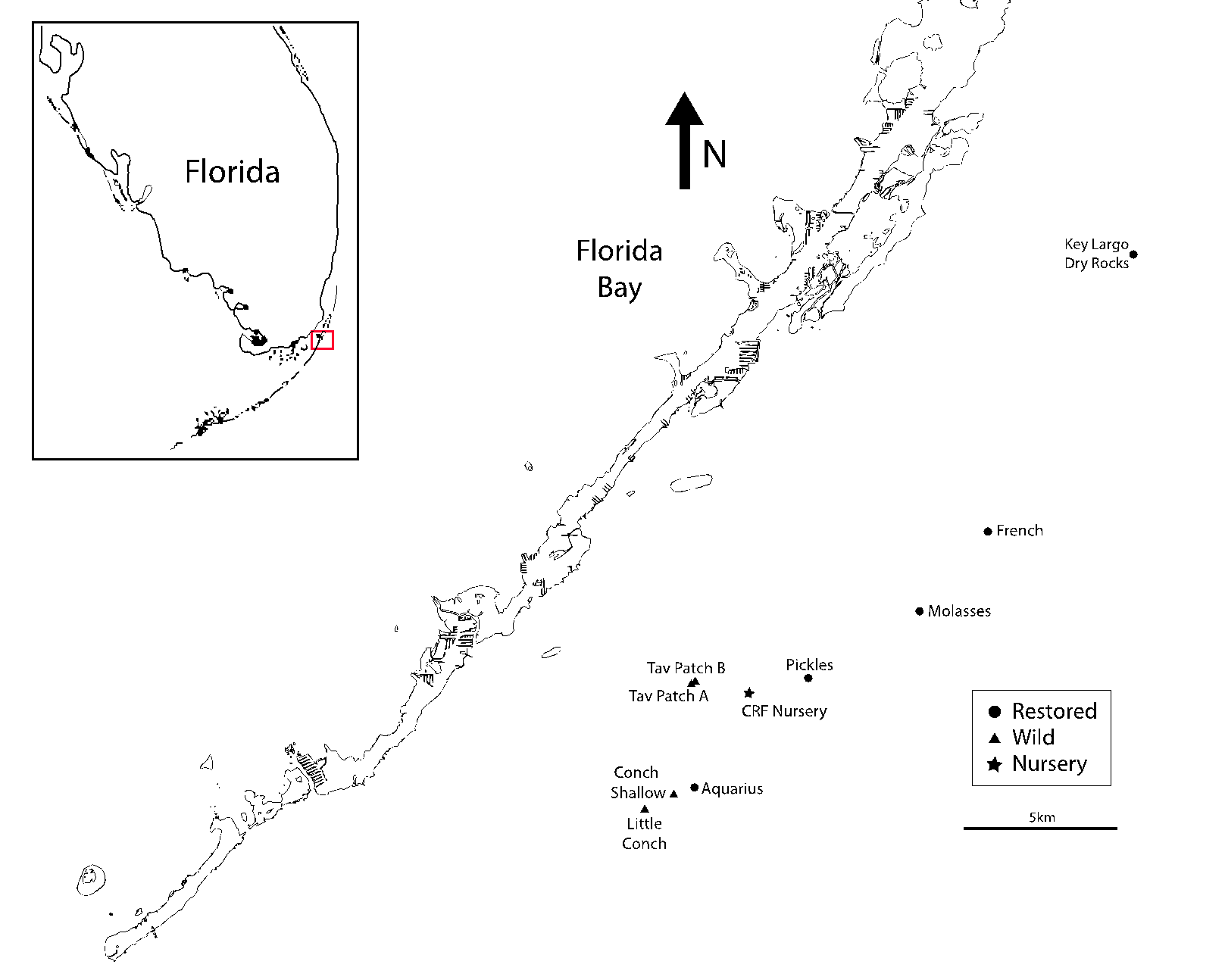

Supplement: Figure S1 — Location of the study sites in the upper Florida Keys. [file peerj-02-541-s005.png]
