# Supplementary material for: Disease dynamics and potential mitigation among restored and wild staghorn coral, Acropora cervicornis
Source: PeerJ. 2014 Aug 28;2:e541. doi: 10.7717/peerj.541 (PMC4157300; doi:10.7717/peerj.541)
Supplement: Supplementary file 5 [file peerj-02-541-s004.docx]

	Epoxy Band	Excision	Control
**No Tissue Loss**	12	10	10
**Tissue Loss**	5	0	11
**Total**	17	10	21
